# Computational and bioactivity investigations of flavonoid fraction from *Dodonaea viscosa* against oxidative stress and inflammation

**DOI:** 10.1038/s41598-025-29576-0

**Published:** 2025-12-11

**Authors:** Amal M. El-Feky, Ahmed A. El-Rashedy, Noha E. Ibrahim

**Affiliations:** 1 Pharmacognosy Department, National Research Centre, 33 El Bohouth St. (Former El Tahrir St.), Dokki, P.O. 12622, Giza, Egypt; 2 Chemistry of Natural and Microbial Products Department, National Research Center (NRC), Giza, Egypt; 3 Department Organic and Medicinal Chemistry, Faculty of Pharmacy, University of Sadat City, Menoufia, 32897 Egypt; 4 Microbial Biotechnology Department, Biotechnology Research Institute, National Research Centre, 33 El Bohouth St. (Former El Tahrir St.), P.O. 12622, Dokki, Giza, Egypt

**Keywords:** *Dodonaea viscosa* leaves, Antioxidant, Anti-inflammatory, Flavonoids, Docking study, Biochemistry, Chemical biology, Chemistry, Drug discovery, Plant sciences

## Abstract

*Dodonaea viscosa* has long been recognized for its medicinal value, yet a comprehensive understanding of its phytochemical composition and bioactivity remains limited. In this study, a flavonoid-enriched fraction from *D. viscosa* leaves was investigated by LC–ESI–MS analysis, revealing six phenolic acids and their derivatives alongside sixteen flavonoids, primarily flavones and flavonols. Quantitative analysis confirmed high levels of flavones and flavonols (2518.6 ± 0.13 mg rutin equivalent/100 g extract), while flavanones and dihydroflavonols were present at significantly lower concentrations (401.2 ± 0.11 mg naringenin equivalent/100 g extract). The extract demonstrated potent antioxidant activity, with DPPH scavenging values of 58.36 ± 18, 76.85 ± 13, and 89.75 ± 19%, and ABTS scavenging values of 69.56 ± 0.06, 76.42 ± 0.10, and 84.28 ± 0.07% at concentrations of 10, 50, and 100 μg/mL, respectively. It also exhibited strong dual inhibitory effects against COX-2 (IC₅₀ = 38.21 μg/mL) and 5-LOX (IC₅₀ = 40.72 μg/mL), comparable to indomethacin (IC₅₀ = 33.03 μg/mL) and zileuton (IC₅₀ = 33.41 μg/mL). Mechanistic insights from tandem mass spectrometry and molecular docking further confirmed key interactions responsible for these bioactivities. Collectively, these findings underscore the therapeutic potential of *D. viscosa* as a natural source of multifunctional bioactive compounds suitable for pharmaceutical and nutraceutical applications. Molecular docking further validated the strong binding affinities of the major flavonoids, with isokaempferide showing particularly stable interactions with COX-2, 5-LOX, and NAD(P)H oxidase. MM-GBSA and hydrogen bond analyses highlighted key residues involved in target inhibition, reinforcing the experimental findings and supporting the potential of *D. viscosa* flavonoids as promising multifunctional therapeutic leads.

**Supplementary Information:**

The online version contains supplementary material available at 10.1038/s41598-025-29576-0.

## Introduction

In recent years, ethno-medicinal research has gained considerable attention, particularly in the exploration of plant-derived bioactive compounds for therapeutic applications. The evaluation of such natural resources necessitates rigorous scientific methodologies, including phytochemical profiling, pharmacological assessments, and clinical validation^1^**.** Despite significant advancements, a comprehensive investigation into the biological activities of flavonoid compounds present in *Dodonaea viscosa* leaves remains limited, particularly through computational approaches^2^**.**

The global resurgence of interest in plant-based therapeutics has spotlighted medicinal flora as reservoirs of bioactive compounds with multifaceted pharmacological potential. Among these, *Dodonaea viscosa*, a perennial shrub of the Sapindaceae family, has garnered attention for its traditional use in treating inflammation, infections, and oxidative stress-related disorders, and is widely distributed across tropical and subtropical regions, It has been employed in ethnomedicine for ailments ranging from rheumatism and ulcers to respiratory and gastrointestinal conditions^3^.

Various parts of the plant are used in traditional medicine: the stems and leaves are employed to treat fever, while seeds—often combined with other botanicals and coated in honey—are used to manage malaria. The leaves are also applied to relieve itching, swelling, aches, and fevers, and are recognized for their antispasmodic properties^4,5^. Trachoma is treated with leaf juice, and powdered leaves are administered to expel roundworms, reflecting the plant’s broad ethnopharmacological relevance^6^**.**

Phytochemical investigations have revealed that *D. viscosa* contains a diverse array of secondary metabolites, including flavonoids, phenolic acids, diterpenoids, saponins, tannins, alkaloids, glycosides, and steroids^7^. Among these, flavonoids such as quercetin, pinocembrin, kaempferol, and apigenin are particularly abundant and have been linked to the plant’s potent antioxidant and anti-inflammatory effects^8^**.** Notably, a chloroform–methanol extract from aerial parts has been shown to inhibit spontaneous contractions of intestinal smooth muscle in isolated rat and guinea-pig ileum in a concentration-dependent manner, supporting its traditional use in gastrointestinal disorders^4^. Furthermore, 3-methoxy flavones derived from quercetin and kaempferol, found in the seeds, bark, inflorescences, and leaves, have demonstrated pronounced antiviral activity against polio-, rhino-, and picorna-viruses^9^. The spasmolytic effects of *D. viscosa* are attributed to the presence of diterpenes, sakuranetin, quercetin, and rutin**,** reinforcing its pharmacological versatility^10^**.**

Biologically, *D. viscosa* exhibits a broad spectrum of pharmacological effects, including antioxidant, anti-inflammatory, antimicrobial, anticancer, antidiabetic, wound healing, antiulcer, and cytotoxic activities^11^**.** For instance, methanolic and chloroform extracts of the leaves have shown significant antibacterial activity against *Staphylococcus aureus* and *Escherichia coli*, while also demonstrating cytotoxic effects on liver and cervical cancer cell lines^11^**.** Flavonoids isolated from the plant have been shown to inhibit COX-2 and 5-LOX enzymes, reduce oxidative stress markers, and modulate inflammatory cytokines, supporting their role in managing chronic inflammatory conditions^3^.

In parallel, in silico approaches such as molecular docking have emerged as powerful tools to elucidate the mechanistic basis of phytochemical bioactivity. Docking studies involving *D. viscosa* constituents have demonstrated strong binding affinities to key inflammatory and neurodegenerative targets, including COX-2, TNF-α, and α-synuclein, suggesting their potential as lead compounds for drug development^12^.

Despite these promising insights, comprehensive profiling of the flavonoid-rich fractions of *D. viscosa* leaves and their integrated antioxidant, anti-inflammatory, and molecular interaction profiles remains limited. Therefore, the primary aim of this work is to isolate, characterize, and evaluate a flavonoid-enriched fraction from *Dodonaea viscosa* leaves for its antioxidant and anti-inflammatory activities through in vitro and in silico approaches, thereby providing insight into its therapeutic potential and mechanisms of action.

## Methods

### Chemicals and reagents

The solvents used in this experiment were obtained from Sigma-Aldrich (Germany). DPPH (2,2-diphenyl-1-picrylhydrazyl hydrate) was purchased from Aldrich Chemie (Germany), and 2,2’-azinobis-(3-ethylbenzothiazoline-6-sulfonic acid) (ABTS) was acquired from Fluka Biochemika, Sigma-Aldrich. Ascorbic acid was also obtained from Sigma-Aldrich Chemie. The cyclooxygenase-2 (COX-2) Inhibitor Screening Kit (Catalog #705,010) and the 5-lipoxygenase (5-LOX) Inhibitor Screening Kit (Catalog #760,700) were purchased from Cayman Chemical (Ann Arbor, MI, USA). Indomethacin and zileuton, used as reference anti-inflammatory drugs, were supplied by Sigma-Aldrich (St. Louis, MO, USA). Rutin (Fluka Biochemika, Sigma-Aldrich) and naringenin (Sigma-Aldrich, Germany) were used as reference standards for the quantification of flavonoid subclasses.

### Plant materials

Leaves of *Dodonaea viscosa* (L.) Jacq. were gathered in April 2023 from the Orman Botanical Garden located in Giza, Egypt. The identification of the plant was confirmed by Mrs. Trease Labib, who serves as the head consultant for plant identification at the Agricultural Ministry, Orman Botanical Garden, Giza, Egypt. After collection, the leaves were meticulously arranged on sheets of non-glossy paper and allowed to air-dry in a shaded, well-ventilated area for several days until they were completely dry. Following this process, the dried leaves were ground into a fine powder. A specimen was submitted to the herbarium of the National Research Centre (NRC) in Cairo, Egypt, under Voucher Number M271.

### Extraction

The dried powdered leaves (250 g) were subjected to separate extraction methods using ethyl alcohol at room temperature. The conventional cold extraction procedure was carried out over three days, repeated five times. The extract obtained was evaporated under reduced pressure using a rotary evaporator (Buchi Rotavapor R-200 combined with a Buchi Vac V-500 pump, Switzerland) at 50 °C, yielding a crude solvent-free extract weighing 10 g**.** To derive a flavonoid-rich fraction, the extract was suspended in 250 mL of water and partitioned with ethyl acetate (2 L, repeated three times), producing a flavonoid-enriched fraction of 7.5 g. This fraction was stored at 4 °C for later phytochemical and biological analyses^13^.

### Quantitative determination of flavones and flavonols

The quantification of flavones and flavonols was conducted following the methodology outlined by^14^. In summary, 1 mL of the ethyl acetate extract was combined with 1 mL of a 2% AlCl3-ethanol solution. Absorbance was measured at 420 nm after the reaction had occurred for 1 h at room temperature. Rutin (Fluka Biochemika, Sigma-Aldrich) was selected as the reference for constructing the calibration curves. The results were expressed as mg of rutin equivalent per 100 g of extract, presented as mean ± S.D.

### Quantitative determination of flavanones and dihydroflavonols

The levels of flavanones and dihydroflavonols have been evaluated following the methodology outlined by^14^. To summarize, 1 mL of the ethyl acetate extract (1 mg/mL in ethanol) was combined with 1 mL of a solution containing 1 g of 2,4-dinitrophenylhydrazine in 2 mL of concentrated sulphuric acid, followed by dilution with methanol to a total volume of 100 mL. The mixture was then heated for 1 h at 45 °C, after which it was diluted with 10% alcoholic KOH to achieve a final volume of 10 mL. Subsequently, 1 mL of the resulting mixture was added to 10 mL of methanol to create a final volume of 50 mL. Absorbance was measured at 486 nm, and the results were presented as mg of naringenin equivalent per 100 g of extract, expressed as mean ± S.D.

### Identification of the flavonoids by LC–ESI–MS technique

The fraction of *Dodonaea viscosa* leaves that is rich in flavonoids was individually analyzed using LC–ESI–MS, in accordance with the procedure outlined by El Feky and Mohammed^15^. The chromatographic separation was performed utilizing an Axion AC system from Kyoto, Japan, which was linked to an autosampler system, an In Line filter disks precolumn (0.5 µm × 3.0 mm, Phenomenex, USA), and an Xbridge C18 (3.5 µm × 2.1 mm × 50 mm) column from Waters Corporation, Milford, MA, USA. The column was kept at a temperature of 40°C, with a flow rate of 300 μL/min utilized. The mobile phase consisted of two solutions: the first solution included 5 mM ammonium formate in 1% methanol, with the pH adjusted to 3.0 using formic acid, while the second solution contained 5 mM ammonium formate in 1% methanol, with a pH of 8.0 attained through the use of ammonium hydroxide. Mass analysis was performed using a Triple TOF 5600 + system equipped with a Duo-Spray source operating in the ESI mode. The sprayer capillary and declustering voltages were set at -4500 V and -80 V, respectively. The temperature was maintained at 600 ◦C. Mass data were generated using the 3.70 software (Yokohama, Kanagawa, Japan). High-resolution survey spectra were confirmed within the m/z range of 40 to 800. The identification of flavonoids was accomplished by correlating their mass spectra with available reports.

### Antioxidant activity

Two distinct antioxidant assays were performed to evaluate the radical scavenging activity of the ethyl acetate fraction from dried *Dodonaea viscosa* leaves. The assays measured the scavenging of α,α-diphenyl-β-picrylhydrazyl (DPPH) and 2,2’-azinobis-(3-ethylbenzothiazoline-6-sulfonic acid) (ABTS) radicals at concentrations of 10, 50, and 100 μg/mL, following the methods described by Rahman et al.^16^**.** Ascorbic acid served as the reference standard^17^. The radical scavenging activity was expressed as the percentage of inhibition, calculated using the following equation^18^:

$$\text{\%}\hspace{0.17em}\text{Inhibition}=\frac{\text{A control}-\text{A sample}}{\text{A control}}x 100$$

where A control is the absorbance of the control reaction and A sample is the absorbance in the presence of the extract or standard.

### In vitro anti-inflammatory evaluation

The anti-inflammatory effects of the ethyl acetate fraction from dried *Dodonaea viscosa* leaves was assessed in vitro using various concentrations (1, 10, 100 μg/mL). This assessment involved measuring COX-2 inhibition with Inhibitor Screening Kits (Milpitas, CA, USA), where absorbance readings were taken at UV-410 nm alongside a blank. Indomethacin was employed as a reference drug for this assay. In addition, an inhibition assay was performed with the human recombinant 5-LOX enzyme using a 5-LOX kit (Sigma-Aldrich), and results were compared to those obtained with Zileuton as a reference drug. Absorbance was measured at UV-490 nm against a blank. The assay methodology was based on the research of El-Feky and El-Rashedy^19^. IC_50_ values were calculated from the inhibition curves using the sigmoid dose–response model and linear regression analysis in Microsoft Office Excel 2010.

### Statistics

All experiments were independently performed in triplicate. Data are expressed as mean ± standard deviation (SD). Statistical analysis was conducted using one-way analysis of variance (ANOVA) to evaluate differences between groups, followed by post hoc comparisons where appropriate. Analyses were carried out using the Statistical Package for the Social Sciences (SPSS, version 11.0 for Windows), and a *p*-value less than 0.05 was considered statistically significant.

#### System preparation and molecular docking

The crystal structures of three target enzymes—Human 5-lipoxygenase (5-LOX, PDB ID: 3V99)^20^, NAD(P)H oxidase from *Lactobacillus sanfranciscensis* (PDB ID: 2CDU)^21^, and Human Cyclooxygenase-2 (COX-2, PDB ID: 5KIR)^22^—were retrieved from the Protein Data Bank. Protein preparation was carried out using UCSF Chimera^23^. Protonation states were optimized at pH 7.5^24^ using PROPKA. The 2D chemical structures of the test compounds were sketched using ChemBioDraw Ultra 12.1^25^, then converted and energy-minimized using Avogadro software^26^**,** employing the MMFF94 force field and the steepest descent algorithm. Prior to docking, all crystallographic water molecules were removed from the protein structures using UCSF Chimera. Polar hydrogens were then added, and Gasteiger charges were assigned to ensure proper protonation and ligand–receptor interactions during docking simulations.

#### Molecular docking

Molecular docking simulations were carried out using AutoDock Vina^27^. Gasteiger partial charges^28^ were applied to the ligands during preparation, and AutoDock-specific atom types^29^ were defined using the AutoDock graphical user interface provided by MGL Tools. Docking grids were generated with center coordinates set as follows: − 11.57, − 10.44, − 71.07 for 5-LOX (PDB ID: 3V99); 2.08, 5.10, 1.69 for NAD(P)H oxidase (PDB ID: 2CDU); and 19.11, − 0.30, 34.54 for COX-2 (PDB ID: 5KIR). For all targets, the grid box dimensions were maintained at 20 Å × 20 Å × 20 Å, with the exhaustiveness parameter set to 8 to ensure adequate sampling. Docked conformations were ranked based on binding affinity, using the Lamarckian Genetic Algorithm^30^ to explore optimal binding poses. Supplementary Table S1 presents the docking scores and interaction profiles of the extracted ligands within the active sites of the target receptors.

#### Molecular dynamic (MD) simulations

Molecular dynamics (MD) simulations offer valuable insights into the time-dependent behavior of biomolecular systems, enabling the exploration of atomic-level motions that are often inaccessible through static experimental techniques^31^. These simulations allow for the detailed investigation of processes such as conformational transitions, molecular interactions, and stability within complex biological environments^32^. In this study, MD simulations were conducted using the GPU-accelerated version of the PMEMD engine within the AMBER 18 software suite^33^**.** For each target protein, the protein–ligand complexes with the most favorable docking interactions (i.e., lowest binding energies and best binding poses) were selected for MD simulations.

Ligand parameters were generated using the General AMBER Force Field (GAFF) via the ANTECHAMBER module^34^, and partial atomic charges were calculated accordingly. Each protein–ligand complex was solvated in an orthorhombic box filled with TIP3P water molecules, extending 10 Å beyond any solute atom. To maintain system neutrality, appropriate numbers of Na⁺ and Cl⁻ counter-ions were added using the LEaP module.

Initial system minimization was performed in two stages: a 2000-step minimization with a restraint of 500 kcal/mol·Å^2^ on the solute, followed by an unrestrained 1000-step conjugate gradient minimization. Subsequently, the systems were gradually heated from 0 to 300 K over 500 ps under constant volume conditions, applying a 10 kcal/mol harmonic restraint on the solute and a 1 ps⁻^1^ collision frequency using a Langevin thermostat.

Following heating, a 500 ps equilibration was carried out at constant temperature (300 K) and pressure (1 bar), employing the Berendsen barostat^35^. The production phase was performed under an isothermal-isobaric (NPT) ensemble with constant pressure, temperature, and randomized velocity seeds. The SHAKE algorithm was applied to constrain all bonds involving hydrogen atoms, allowing for a 2 fs time step. The SPFP precision model was used throughout the simulations for enhanced accuracy and performance.

#### Post-MD analysis

Following the completion of molecular dynamics (MD) simulations, system trajectories were saved at 1 ps intervals and subsequently analyzed using the CPPTRAJ module integrated within the AMBER18 software suite^36^. Structural and dynamic parameters were extracted from these trajectories to evaluate the stability and conformational behavior of the protein–ligand complexes. Graphical representations and three-dimensional visualizations were generated using Origin and UCSF Chimera (Pettersen et al., 2024), respectively, to support the analysis and interpretation of the simulation data.

#### Thermodynamic calculation

To estimate the binding affinities of ligands to their respective protein targets, the Molecular Mechanics/Poisson–Boltzmann Surface Area (MM/PBSA) and Molecular Mechanics/Generalized Born Surface Area (MM/GBSA) methodologies were employed. These approaches, based on continuum solvation models, provide an efficient means to evaluate the free energy of binding using molecular dynamics trajectories^37^**.** Binding free energies were computed by averaging data from 200 snapshots extracted evenly across the 20 ns simulation period. The calculations incorporated both gas-phase interaction energies (including van der Waals, electrostatic, and internal energy components) and solvation effects^38^. The latter were divided into polar and non-polar contributions, with the polar solvation energy estimated using the Generalized Born (GB) model and the non-polar component derived from solvent-accessible surface area (SASA) calculations, applying a water probe radius of 1.4 Å.

Entropy contributions were considered via the temperature and solute entropy terms, although entropic calculations may vary in accuracy and were applied with consideration of their limitations. Additionally, per-residue energy decomposition was conducted using the MM/GBSA module in AMBER18 to identify key amino acid residues contributing to ligand binding^39^**.**

## Results

### Quantitative determination of flavonoid subclasses

**Fig. 1:**
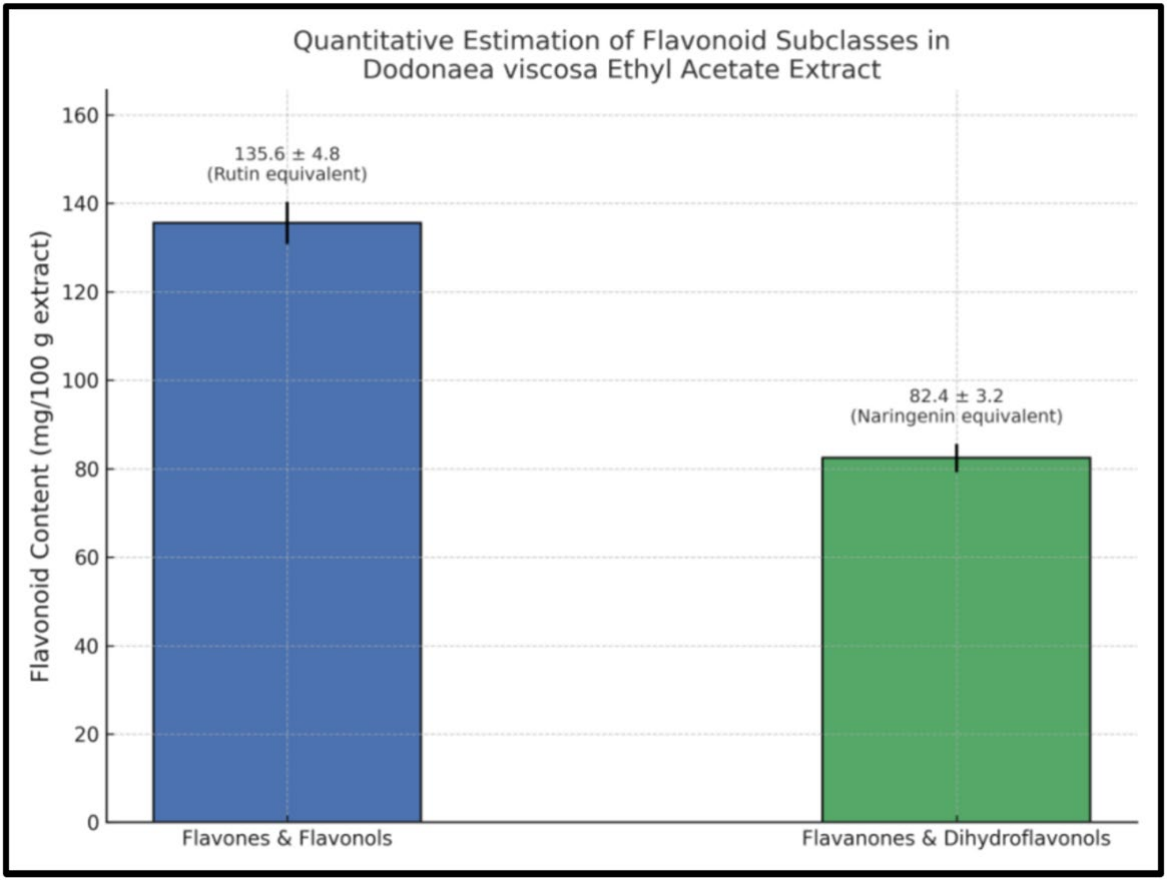
Quantitative determination of flavonoid subclasses in the ethyl acetate extract of *Dodonaea viscosa* leaves.

The ethyl acetate extract of *Dodonaea viscosa* leaves was found to be rich in flavonoid content. Quantitative analysis revealed that the concentration of flavones and flavonols was 135.6 ± 4.8 mg rutin equivalent (RE)/100 g extract, while flavanones and dihydroflavonols were present at 82.4 ± 3.2 mg naringenin equivalent (NE)/100 g extract. These values confirm that the ethyl acetate fraction is notably enriched with flavonoid subclasses known for their pharmacological relevance. The substantial presence of these compounds supports the selection of this fraction for further antioxidant and anti-inflammatory evaluations. The comprehensive results illustrated in Fig. 1.

### Identification of the flavonoids by LC–ESI–MS technique

**Fig. 2:**
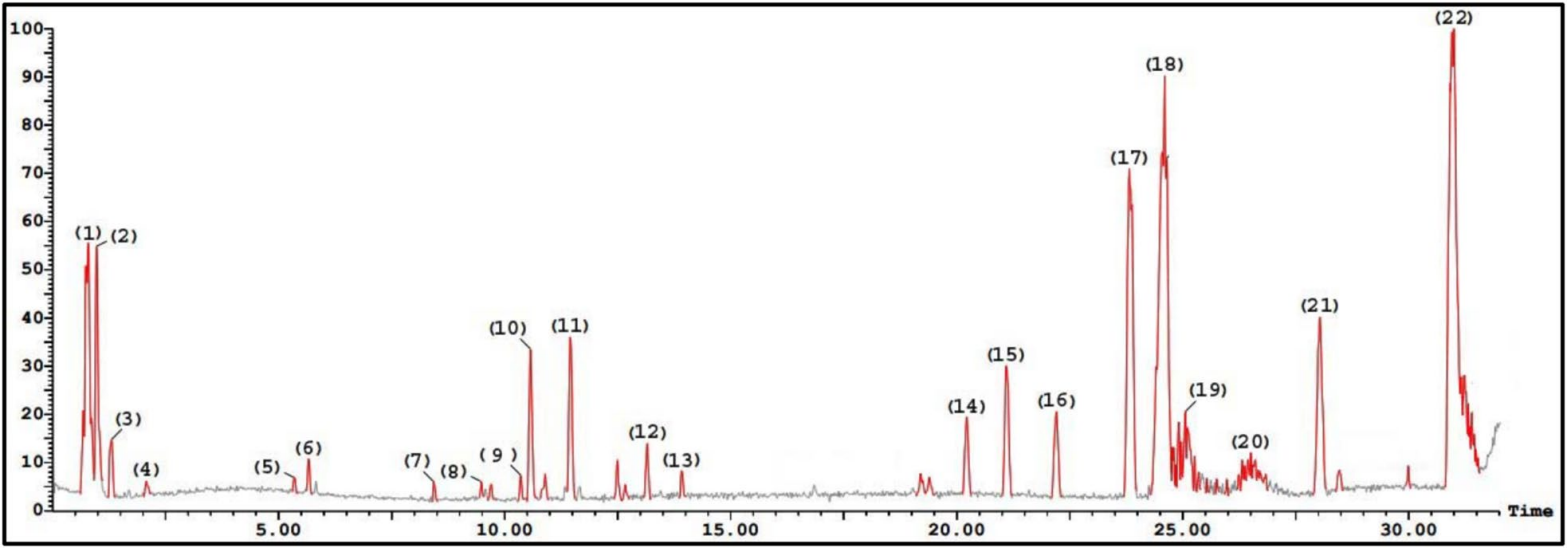
UPLC/ESI–MS chromatogram of polyphenolic compounds in *Dodonaea viscosa* leaves in negative ion mode.

Liquid chromatography–electrospray ionization mass spectrometry (LC–ESI–MS) was employed to characterize the major phytochemical constituents within the flavonoid-enriched fraction of *Dodonaea viscosa* leaves. The analysis yielded a detailed profile of the detected compounds, including retention times (Rt), deprotonated molecular ions [M − H]⁻, and corresponding MS/MS fragment ions, as summarized in Table 1. Representative chromatographic profiles of these compounds are presented in Fig. 2.

**Table 1:** Characterization of polyphenolic compounds in *Dodonaea viscosa* leaves using LC ESI–MS/MS in negative ion mode.

Peak No	Retention Time (min)	Tentative identification	M.W	Experimental m/z [M − H]^−^	MS/MS fragments (m/z)	Chemical Class	References
1	0.79	*P*-Coumaric acid	164	163.0611	145[M-H_2_O],119[M-H-CO_2_]	Hydroxycinnamic acid	^9^
2	0.97	Feruloylquinic acid	367	368.1371	193[M-H-feruloyl],173[M-H-feruloyl- H_2_O]	hydroxycinnamic acid ester	
3	1.30	Caffeoyl-*O*-hexoside	342	341.2715	179[M-H-caffoeyl],161[M-H- caffoeyl-H_2_O],135[M-H-caffoeyl- CO_2_]	Hydroxycinnamic acid glycoside	
4	2.08	Chlorogenic acid	354	353.1088	191, 173, 119	Hydroxycinnamic acid	^40^
5	5.36	p-Coumaric acid ethyl ester	192	191.0573	147[M-H–CO₂], 118[M-H–CO₂-C₂H₅], 174 [M-H-OH]	Cinnamate ester	^41^
6	5.66	Myricetin-*O*-hexoside	480	479.3819	461[M-H-H_2_O],317[M-H -hexoside] ,299[M-H-Hexoside -H_2_O], 179, 151	Flavonol glycoside	
7	8.44	Coumaroyl-*O*-caffeoylquinic acid	500	499.2168	353[M-H-coumaroyl],337[M-H caffoey], 325[M-H-quinic acid-H_2_O] 191[quinic acid],179[caffiec acid]	Hydroxycinnamic acid ester	
8	9.48	Quercetin-*O*- hexoside	464	463.0857	301[M-H- hexoside], 271[M-H- hexoside -CH_2_O], 257, 255[M-H- hexoside-CO-H_2_O], 179	Flavonol glycoside	
9	10.36	5,7,4-Trihydroxy-3-(4-hydroxy 3-Methylbutyl)-5-prenyl-3, 6-diMethoxyflavone	484	483.2186	453,331, 397	Flavone	^41^
10	10.57	Kaempferol-*O*-rutinoside	594	593.1486	285[M-H-rutinoside], 255[M-H-rutinoside-CH_2_O],227[M-H-rutinoside-2CHO], 179, 151	Flavonol glycoside	
11	11.45	Quercetin-*O*-pentoside	434	433.2204	301[M-H- pentoside],271[M-H- pentoside-CH_2_O],257, 179	Flavonol glycoside	
12	13.16	Viscosol	412	411.2415	396, 331,265	Flavone	^42^
13	13.91	Catechin	578	577.1333	435, 425, 407, 289, 245, 205, 179, 151	Flavan-3-ol	^6^
14	20.22	Rutin	610	609.1437	301[M-H-rutinoside],271[M-H-rutinoside -CH_2_O],257, 255[M-H-rutinoside-CO-H_2_O],179,151	Flavonol glycoside	Hamadi, 2017
15	21.09	Kaempferol-*O*-rhamnoside	432	431.1357	387 ,285[M-H-rhmnoside],161, 179, 151	Flavonol glycoside	
16	22.21	Quercetin 3’ -*O*-methyl ether	316	315.1465	315, 293, 316, 249, 287	Flavonol glycoside	^40^
17	23.82	3,5-Dihydroxy-4’ ,7-dimethoxyflavone	315	313.2390	283, 255, 161	Flavone	^43^
18	24.60	5,7,4’ ,5’ -Tetrahydroxy-3,6,2’– trimethoxyflavone	376	375.1685	361, 351, 551	Flavone	^40^
19	25.06	5,7-Dihydroxy-3,6,4’-trimethoxyflavone	344	343.2153	313, 301,270	Flavone	^43^
20	26.50	Kaempferol	286	285.2094	267[M-H-H_2_O],257[M-H–CO], 227 [M-H-2CHO], 179, 151	Flavonol	^44^
21	28.03	Isokaempferide	300	299.1053	375, 347	Flavone	^45^
22	31.00	Quercetin	302	301.2363	273[M-H–CO], 257 [M-H- CO₂], 255[M-H–CO-H_2_O], 151,179	Flavonol	^6^

The phenolic composition of the flavonoid-rich fraction from *Dodonaea viscosa* leaves was examined, revealing the presence of six phenolic acids and their derivatives, along with sixteen flavonoids. The identified phenolic acids and derivatives corresponded to peaks 1–5 and 7, including *p*-coumaric acid, feruloylquinic acid, caffeoyl-*O*-hexoside, chlorogenic acid, *p*-coumaric acid ethyl ester, and coumaroyl-*O*-caffeoylquinic acid. To provide a clearer understanding of their structural characteristics, the fragmentation behavior of these compounds was examined through LC–ESI–MS/MS. Phenolic acids and their esterified derivatives typically experience fragmentation processes such as dehydration (loss of H₂O) and decarboxylation (loss of CO₂), which are enhanced by the presence of reactive functional groups^46^. For example, *p*-coumaric acid (peak 1, R_*t*_ 0.79) displayed a deprotonated molecular ion ([M − H]⁻) at *m/z* 163, which subsequently underwent decarboxylation to produce a fragment ion at *m/z* 119. Furthermore, the cleavage of the hydroxyl group led to the generation of a fragment at *m/z* 145.

Generally, esters and phenolic acids can experience fragmentation pathways that involve the loss of water (H₂O) or carbon dioxide (CO₂). For phenolic acids, decarboxylation (the loss of CO₂) is a prevalent fragmentation pathway, particularly under conditions of electron ionization mass spectrometry. Likewise, dehydration (the loss of H₂O) may occur under specific circumstances, especially when the molecule contains hydroxyl groups that promote this process^46^. This is a clear pathway in *p*-coumaric acid (peak 1, R_*t*_ 0.79), where the deprotonated molecular ion (M − H)⁻ at *m/z* 163 acts as the precursor ion, which then undergoes fragmentation pathways such as decarboxylation (loss of CO₂, 44 Da), resulting in a fragment at *m/z* 119. Furthermore, the cleavage of the hydroxyl (-OH) group can yield a fragment at *m/z* 145.

Feruloylquinic acid (peak 2, R_t_ 0.97), a derivative of hydroxycinnamate, exhibits distinctive fragmentation patterns in negative mode liquid chromatography-mass spectrometry (LC–MS). Upon ionization, it generates significant fragment ions as a result of ester bond cleavage and decarboxylation. The fragmentation pathways reveal the loss of ferulic acid and quinic acid, producing diagnostic ions that facilitate structural identification. The detection of *m/z* 193, which corresponds to ferulic acid, and *m/z* 173, linked to the rearrangement of quinic acid, is frequently noted.

Chlorogenic acid (peak 4, R_*t*_ 2.08), a polyphenolic compound commonly found in coffee and various plants, exhibits characteristic fragmentation when analyzed using negative mode LC–MS. Upon ionization, it readily generates *m/z* 191, which corresponds to quinic acid, and *m/z* 179, which is indicative of caffeic acid. The cleavage of the ester bond between caffeic and quinic acid represents a primary pathway, while additional fragmentation can produce ions such as *m/z* 135, linked to the degradation of caffeic acid, and *m/z* 173, resulting from the rearrangement of quinic acid. These fragment ions act as diagnostic markers in LC–MS/MS analysis^47^**.**

*P*-Coumaric acid ethyl ester [M − H]^−^ 191, (peak 5), a derivative of *p*-coumaric acid, undergoes decarboxylation (loss of CO₂, 44 Da), resulting in the product ion *m/z* 147. The neutral loss of the ethyl group (-C₂H₅, 29 Da) from the decarboxylated fragment (147 Da) leads to a mass of 118 Da, which indicates the cleavage of its ester bond. Furthermore, phenolic acids frequently exhibit fragmentation pathways that involve the loss of hydroxyl (-OH) groups (-OH, 17 Da) from the original molecule (191 Da). The mass of the fragment was 174 Da, contributing to diagnostic ions that assist in structural identification.

Coumaroyl-*O*-caffeoylquinic acid, a complex derivative of hydroxycinnamate, peak7, *m/z* 499, demonstrates fragmentation pathways that involve the cleavage of ester bonds, resulting in diagnostic ions that correspond to caffeoylquinic acid and coumaroyl moieties. The fragmentation of the caffeoylquinic component results in *m/z* 191, which represents quinic acid, and *m/z* 179, which is associated with caffeic acid. Further degradation yields *m/z* 135, which is linked to the breakdown of caffeic acid. In a similar manner, the coumaroyl moiety produces significant fragments such as *m/z* 163, which is indicative of *p*-coumaric acid, and *m/z* 119, which is a product of its degradation.

Multiple phenolic acids that conjugate with glycosides generate several common fragmentation ions during MS/MS analysis, specifically through the loss of a sugar moiety, as illustrated in peak 3. Caffeoyl-*O*-hexoside (R_*t*_ 1.30), which is a glycosylated derivative of caffeic acid, exhibits characteristic fragmentation. The deprotonated molecular ion (M − H)⁻ generally appears at *m/z* 341, which corresponds to the complete caffeoyl-hexoside structure. When fragmentation occurs, the cleavage of the glycosidic bond results in the formation of m/z 179, which represents caffeic acid, a crucial diagnostic ion. Further fragmentation of caffeic acid leads to the production of *m/z* 135, which is linked to the loss of carboxyl and hydroxyl groups. Additional rearrangements and neutral losses can produce fragments at *m/z* 134 and *m/z* 126, which assist in structural identification.

Additionally, the analysis uncovered the presence of different classes of flavonoids, including flavones, flavan-3-ol, flavonols, and flavonol glycosides. Flavones, which are a subclass of flavonoids, demonstrate distinct fragmentation patterns, mainly through retro-Diels–Alder (RDA) reactions, along with neutral losses of CO₂ and CH₃ radicals. The flavones identified include 5,7,4-trihydroxy-3-(4-hydroxy 3-methylbutyl)-5-prenyl-3,6-dimethoxyflavone (peak 9, R_t_ 10.36), viscosol (peak 12, R_*t*_ 13.16), and peaks 17–19, which correspond to 3,5-dihydroxy-4’,7-dimethoxyflavone, 5,7,4’,5’-tetrahydroxy-3,6,2’–trimethoxyflavone, and 5,7-dihydroxy-3,6,4’-trimethoxyflavone (R_*t*_ 23.82, 24.60, and 25.06, respectively), in addition to isokaempferide (peak 21, R_*t*_ 28.03). Their identification is based on their fragmentation behavior. These compounds generally experience the loss of methoxy (-OCH₃) and hydroxyl (-OH) groups, resulting in diagnostic ions that facilitate structural elucidation. Hydroxyalkyl substitutions may present unique fragmentation pathways, producing ions at *m/z* 153, *m/z* 167, and *m/z* 151, which are essential for differentiation^48^**.**

Furthermore, the analysis indicated the presence of catechin as a flavan-3-ol (peak 13, R_*t*_ 13.91), along with two flavonols identified as kaempferol and quercetin (R_*t*_ 26.50 and 31.00, respectively). The deprotonated molecular ion (M − H)⁻ of catechin at *m/z* 577 implies the existence of a dimeric or conjugated catechin structure, which experiences fragmentation pathways including the cleavage of interflavonoid bonds, neutral loss of hydroxyl (-OH) groups, and retro-Diels–Alder (RDA) reactions^49^. The fragmentation of catechin results in significant ions at *m/z* 289, which corresponds to the monomeric catechin unit, and *m/z* 245, which is related to the loss of a carbonyl group. Additional fragmentation leads to *m/z* 205, associated with the loss of hydroxyl groups, while further rearrangements produce fragments at *m/z* 179 and *m/z* 151, which assist in structural identification.

Moreover, kaempferol and quercetin, two extensively researched flavonols, display distinct fragmentation patterns. The deprotonated molecular ion (M − H)⁻ is observed at *m/z* 285 for kaempferol and at *m/z* 301 for quercetin. During fragmentation, both substances experience retro-Diels–Alder (RDA) cleavage, resulting in diagnostic ions such as *m/z* 151, which is indicative of the A-ring, and *m/z* 179, which corresponds to the loss of the B-ring. Quercetin, owing to its extra hydroxyl group, frequently yields *m/z* 257, which arises from the neutral loss of CO₂. Research suggests that flavonols containing methoxy groups demonstrate radical losses of CH₃•, while hydroxylated flavonols produce fragments at *m/z* 153 and *m/z* 167, which assist in structural elucidation^50^**.**

In this research, various flavonol glycosides were detected based on their retention times (R_*t*_) during LC–MS analysis, such as Kaempferol-*O*-rutinoside (R_*t*_ 10.57) and Kaempferol-*O*-rhamnoside (R_t_ 21.09), which are derivatives of kaempferol associated with sugar moieties. Their fragmentation pathways involve the loss of sugar moieties, resulting in the formation of the aglycone kaempferol (*m/z* 285). Further fragmentation of kaempferol leads to *m/z* 151, which is indicative of the A-ring, and *m/z* 179, which corresponds to the loss of the B-ring^51^**.**

Quercetin glycosides, including quercetin-*O*-hexoside (R_*t*_ 9.48), quercetin-*O*-pentoside (R_*t*_ 11.45), and Rutin (R_*t*_ 20.22), follow comparable fragmentation pathways, characterized by the loss of sugar moieties that result in quercetin (*m/z* 301). The fragmentation of quercetin yields *m/z* 257, which arises from the neutral loss of CO₂, and *m/z* 179, which is associated with the cleavage of the B-ring^52^. Furthermore, Quercetin 3’-*O*-methyl ether (R_*t*_ 22.21) displays fragmentation patterns that involve the loss of the methyl (-CH₃) group, leading to the generation of *m/z* 287, which is beneficial for structural elucidation. Myricetin glycosides, such as myricetin-*O*-hexoside (R_t_ 5.66), exhibit fragmentation pathways that are analogous to those of quercetin derivatives, with the loss of sugar moieties resulting in myricetin (*m/z* 317). Additional fragmentation yields *m/z* 179, which indicates B-ring cleavage, and *m/z* 151, which corresponds to the A-ring^53^.

### Biological investigation

The dried leaves of *Dodonaea viscosa* are rich in phytochemical constituents that demonstrate a variety of biological activities, including antioxidant, hypoglycemic, and anticancer properties, among others^54,55^. Importantly, phenolic and flavonoid compounds are crucial in the regulation of numerous physiological functions, such as cell proliferation, enzymatic activities, cellular redox balance, and signal transduction pathways, thus playing a vital role in the prevention of chronic diseases^56,57^.

#### Antioxidant

A comparative study was conducted to evaluate the free radical scavenging activity of the flavonoid-rich fraction from the leaves of *Dodonaea viscosa*, specifically against DPPH and ABTS radicals at serial concentrations of 10, 50, and 100 μg/mL, as detailed in Table 2. The results indicated that the leaves of *Dodonaea viscosa* exhibited improved scavenging abilities, with measured values of 58.36 ± 18, 76.85 ± 13, and 89.75 ± 19 for DPPH and 69.56 ± 0.06, 76.42 ± 0.10, and 84.28 ± 0.07 for ABTS radicals, respectively, at concentrations of 10, 50, and 100 μg/ml.

**Table 2:** The free radicals scavenging activity of flavonoids rich fraction from *Dodonaea viscosa* leaves against DPPH and ABTS.

Tested group	DPPH				ABTS			
	Inhibition percentages (%)			IC50	Inhibition percentages (%)			IC50
	10 μg/mL	50 μg/mL	100 μg/mL		10 μg/mL	50 μg/mL	100 μg/mL	
Flavonoid fraction	58.36 ± 18^a^	76.85 ± 13^b^	89.75 ± 19^a^	31.21	69.56 ± 0.06^d^	76.42 ± 0.10^e^	84.28 ± 0.07^d^	27.44
Ascorbic acid	65.32 ± 0.24^c^	83.12 ± 0.09^d^	91.34 ± 0.08^d^	25.83	73.10 ± 0.11^e^	81.96 ± 0.13^b^	89.36 ± 0.11^e^	22.73

Values are represented by mean ± SD of three replicate. The different superscript letters between groups are significant differences at P values < 0.05.

#### In vitro anti-inflammatory effect

The flavonoid-rich extract from the leaves of *Dodonaea viscosa* was evaluated for its anti-inflammatory efficacy by measuring its inhibitory action on two key enzymes: cyclooxygenase-2 (COX-2) and 5-lipoxygenase (5-LOX). According to the data presented in Table 3, the extract exhibited substantial inhibition of COX-2, with an IC₅₀ value of 38.21 μg/mL, which is comparable to the IC₅₀ of the standard reference drug indomethacin (33.03 μg/mL). Additionally, the same extract suppressed the activity of 5-LOX, yielding an IC₅₀ of 40.72 μg/mL, closely aligning with that of zileuton, a well-established 5-LOX inhibitor (IC₅₀ = 33.41 μg/mL). These findings highlight the dual enzyme inhibitory potential of the extract and underscore its promise as a naturally derived anti-inflammatory agent.

**Table 3:** In vitro anti-inflammatory activities of flavonoids rich fraction from *Dodonaea viscosa* leaves against COX-2 and 5-LOX.

Ethyl acetate fraction	COX-2				5-LOX			
	Inhibition (%)			IC50	Inhibition (%)			IC50
	1 μg/mL	10 μg/mL	100 μg/mL		1 μg/mL	10 μg/mL	100 μg/mL	
Flavonoid fraction	37.84 ± 0.12^a^	48.93 ± 0.09^b^	86.98 ± 0.13^a^	38.21	34.98 ± 0.10^c^	47.96 ± 0.08^b^	84.92 ± 0.12^a^	40.72
Indomethacin	43.16 ± 0.08^b^	49.62 ± 0.10^c^	93.1 ± 0.08^b^	33.03				
Zileuton					39.11 ± 0.14^d^	51.26 ± 0.13^a^	94.03 ± 0.09^b^	33.41

Data are mean ± SD of 3 replicates, different superscript letters between groups are significant differences at P values < 0.05.

### Molecular dynamics and system stability

The conformational stability of both the unbound (apo) and ligand-bound protein systems was evaluated over a 100 ns molecular dynamics (MD) production run to ensure the reliability of the simulation and to minimize potential artifacts. System stability was assessed by monitoring the root mean square deviation (RMSD) of the protein backbone atoms throughout the simulation trajectory.

**Fig. 3:**
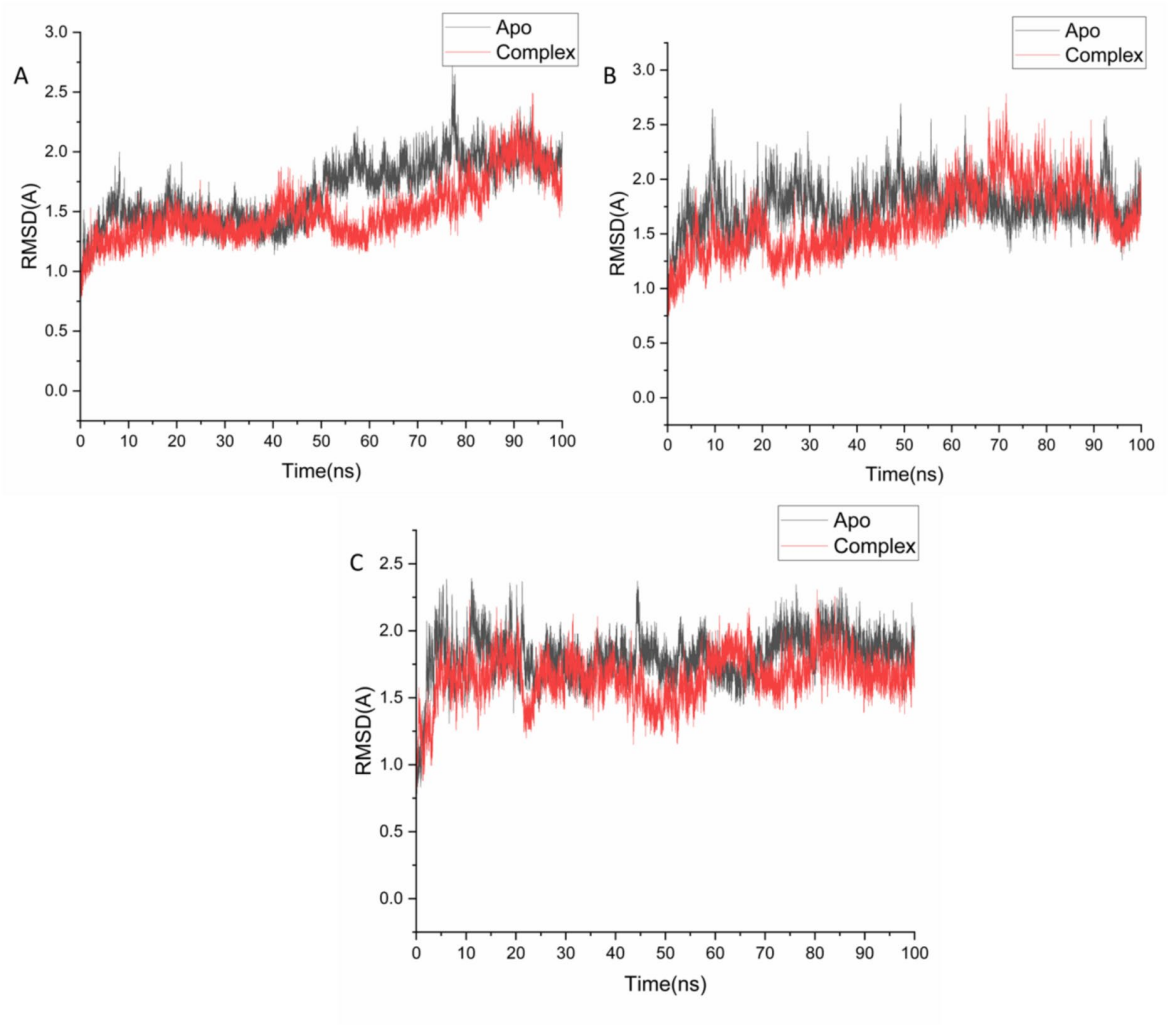
RMSD of Cα atoms of the protein backbone atoms showing the degree of stability upon Isokaempferide binding 5-LOX[A], NAD(P)H [B] and COX-2 [C].

For the 5-lipoxygenase (5-LOX) system, the average RMSD was 1.66 ± 0.26 Å for the apo form and 1.50 ± 0.24 Å for the 5-LOX–Isokaempferide complex (Fig. 3A), indicating minimal structural deviation upon ligand binding. The NAD(P)H oxidase system exhibited RMSD values of 1.75 ± 0.20 Å for the apo structure and 1.63 ± 0.30 Å for the NAD(P)H– Isokaempferide complex (Fig. 3B), suggesting slightly enhanced stability in the presence of the ligand. Notably, the COX-2 system showed a higher deviation in the apo state (1.80 ± 0.17 Å), which was significantly reduced in the COX-2– Isokaempferide complex (1.65 ± 0.17 Å), reflecting a stabilizing effect upon ligand binding (Fig. 3C).

These findings indicate that the binding of Isokaempferide contributes to enhanced conformational stability in all three protein systems when compared to their respective apo forms. The consistently lower RMSD fluctuations observed in the ligand-bound complexes suggest that Isokaempferide establishes stable interactions with 5-LOX, NAD(P)H oxidase, and COX-2, effectively maintaining the structural integrity of the proteins throughout the simulation period. This improved stability reinforces the reliability of subsequent analyses related to binding interactions and dynamic behavior.

**Fig. 4:**
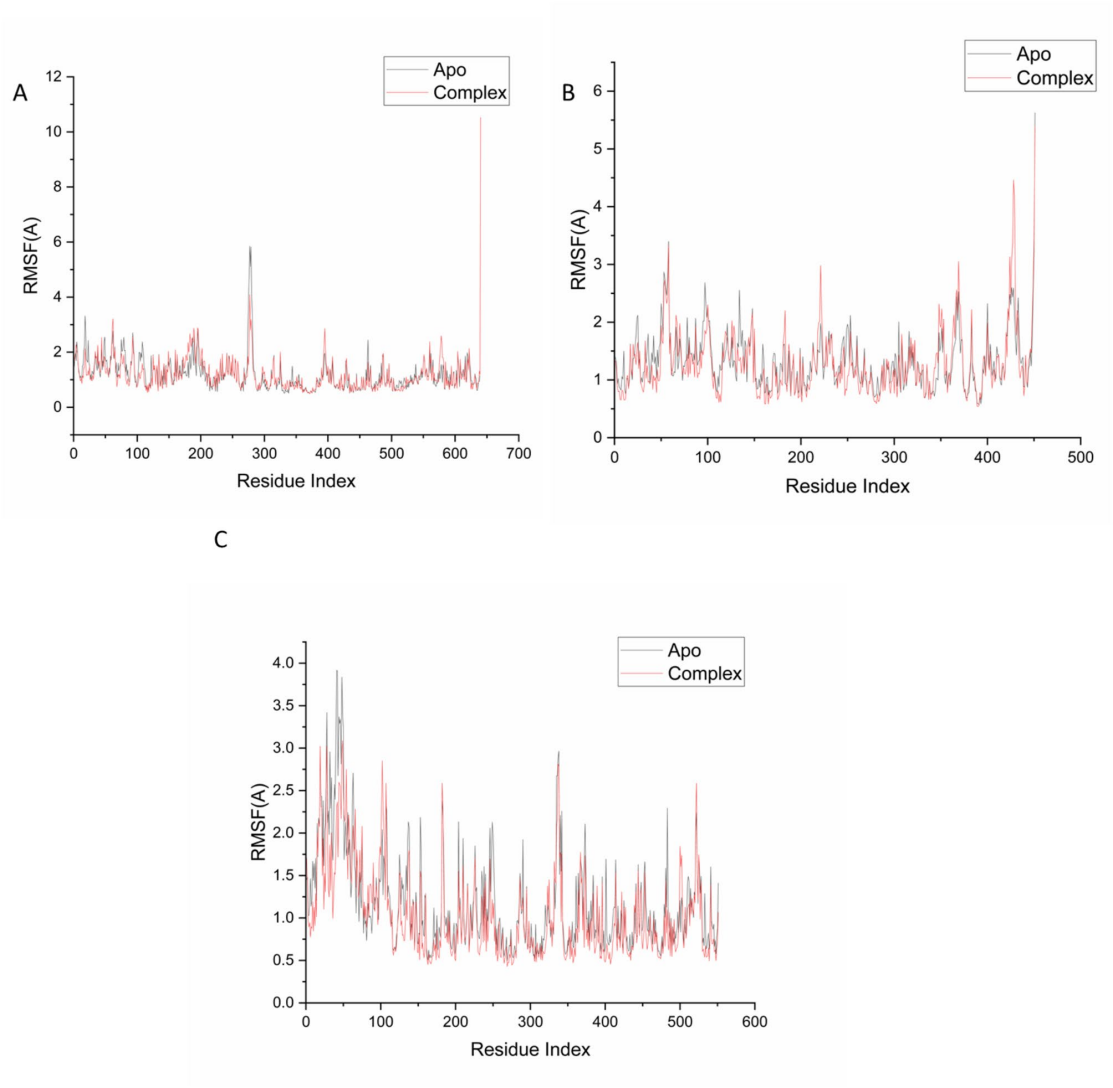
Root mean square fluctuation (RMSF) of Cα atoms showing residue-wise flexibility in the presence of Isokaempferide. The plots compare the apo and ligand-bound forms of (**A**) 5-LOX, (**B**) NAD(P)H oxidase, and (**C**) COX-2, highlighting changes in backbone mobility upon ligand binding.

Furthermore, the binding of Isokaempferide to the target proteins induced localized structural perturbations, as indicated by changes in backbone atom mobility relative to their unbound counterparts. Root mean square fluctuation (RMSF) analysis was performed to assess residue-level flexibility throughout the simulation. For the 5-LOX system, the average RMSF was 1.20 ± 0.60 Å in the apo form and 1.18 ± 0.63 Å in the Isokaempferide-bound complex (Fig. 4A), indicating a slight stabilization upon ligand binding. Similarly, NAD(P)H oxidase showed average fluctuations of 1.32 ± 0.50 Å for the apo form and 1.28 ± 0.57 Å for the complex with Isokaempferide (Fig. 4B). Notably, COX-2 exhibited a more pronounced reduction in atomic fluctuations, with RMSF values of 1.17 ± 0.59 Å for the apo structure and 1.03 ± 0.03 Å for the Isokaempferide-bound complex (Fig. 4C).

Overall, the reduced RMSF values in the ligand-bound forms, particularly in COX-2, suggest that Isokaempferide binding confers increased local stability and dampens residue flexibility, supporting its potential as a stabilizing ligand.

**Fig. 5:**
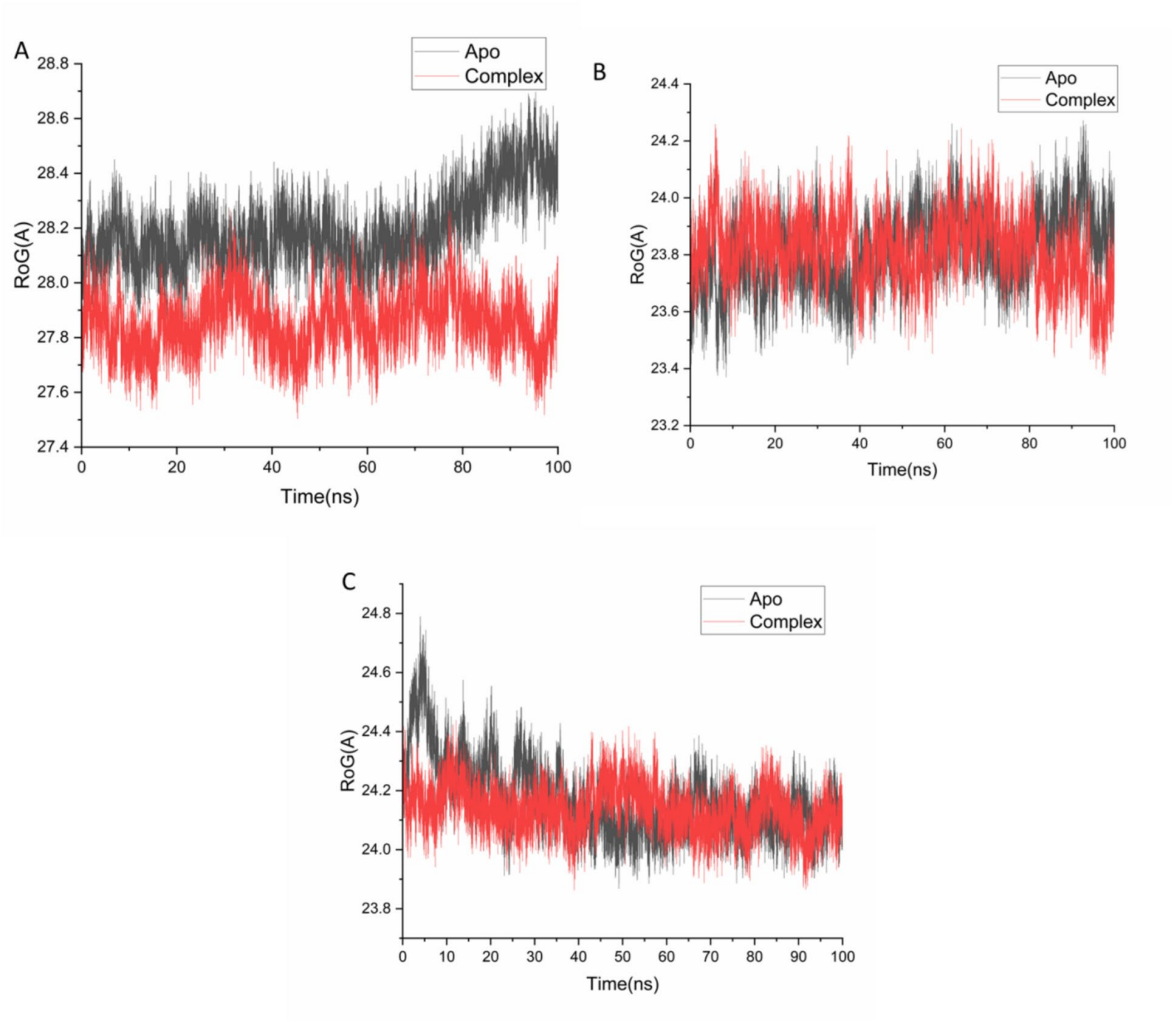
Radius of gyration (Rg) of Cα atoms illustrating the overall structural compactness of the proteins upon Isokaempferide binding. The plots compare the apo and ligand-bound states of (**A**) 5-LOX, (**B**) NAD(P)H oxidase, and (**C**) COX-2, indicating the effect of ligand binding on protein folding and stability.

To evaluate the global structural compactness of 5-LOX, NAD(P)H oxidase, and COX-2 upon ligand binding, the radius of gyration (Rg) was calculated. This parameter reflects the mass-weighted root-mean-square distance of atoms from the protein’s center of mass, serving as an indicator of overall protein folding and compactness^58^**.** The average Rg values were as follows: 28.19 ± 0.13 Å for apo-5-LOX and 27.85 ± 0.10 Å for the 5-LOX– Isokaempferide complex; 23.80 ± 0.12 Å for apo-NAD(P)H and 23.81 ± 0.11 Å for the NAD(P)H– Isokaempferide complex; and 24.16 ± 0.12 Å for apo-COX-2 and 24.13 ± 0.07 Å for the COX-2– Isokaempferide complex, as illustrated in Figs. 5 A–C. The slight reductions in Rg values upon ligand binding suggest an increase in protein compactness, indicating that Isokaempferide may enhance the structural rigidity of the binding region in each target protein. This behavior supports the hypothesis that Isokaempferide forms stable and specific interactions within the catalytic sites.

**Fig. 6:**
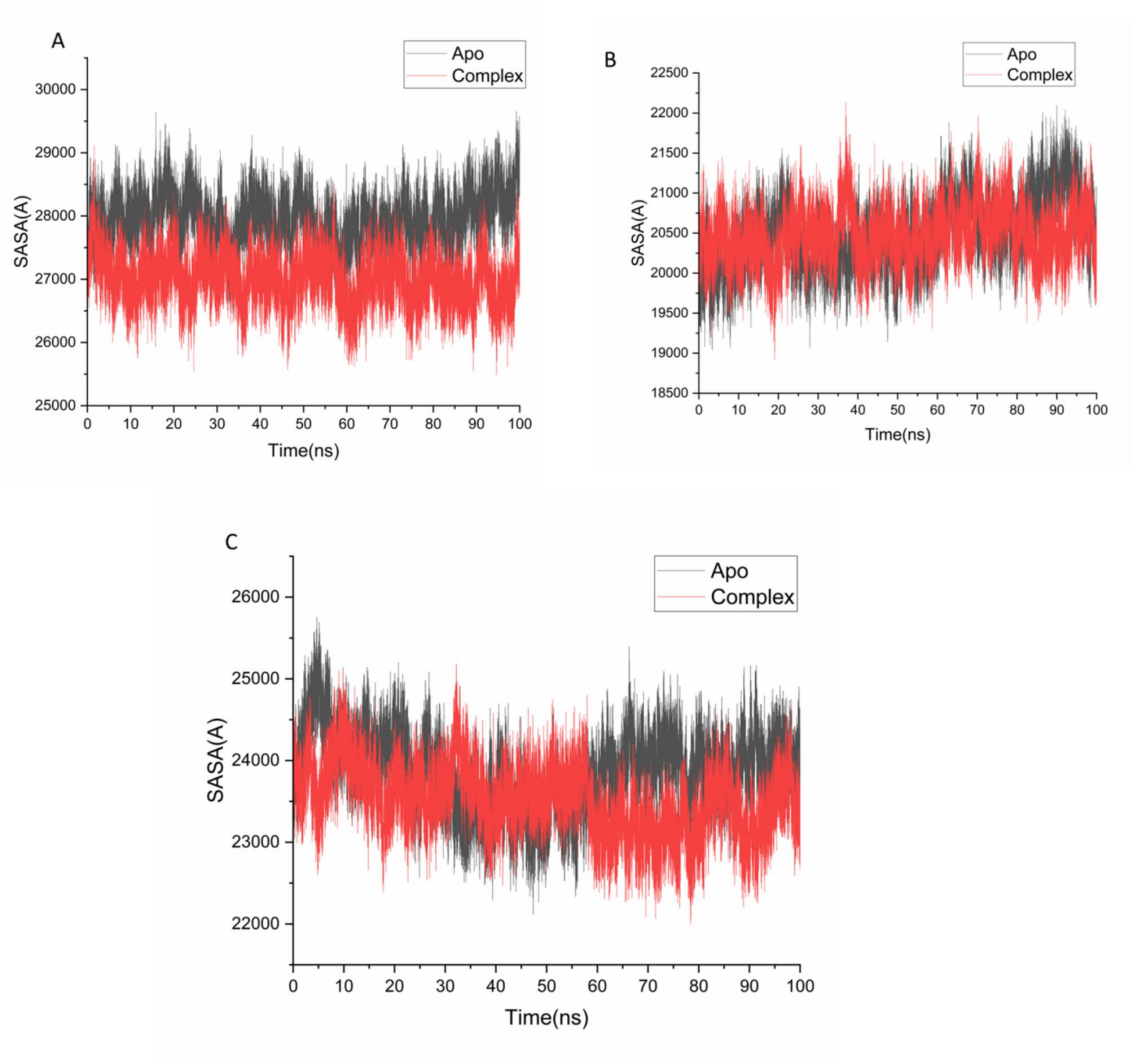
Solvent-accessible surface area (SASA) of backbone atoms over the 100 ns MD simulation, showing changes relative to the initial minimized structure. The plots compare the apo and Isokaempferide-bound states of (**A**) 5-LOX, (**B**) NAD(P)H oxidase, and (**C**) COX-2, highlighting the effect of ligand binding on solvent exposure and structural compactness. Presumably, the observed perturbations in the backbone atoms of 5-LOX, NAD(P)H oxidase, and COX-2 may reflect the mechanistic basis of Isokaempferide’s inhibitory activity, as ligand-induced disruption of structural integrity often correlates with impaired protein function..

To further investigate the structural consequences of ligand binding, solvent-accessible surface area (SASA) analysis was performed to assess protein–solvent interactions and changes in the exposure of hydrophobic regions. SASA quantifies the surface area of the protein that is accessible to solvent molecules, thereby providing valuable insights into protein folding, stability, and compactness^59^. The average SASA values were as follows: 27,961.73 Å^2^ for apo-5-LOX and 26,997.41 Å^2^ for the 5-LOX– Isokaempferide complex; 20,483.87 Å^2^ for apo-NAD(P)H and 20,474.36 Å^2^ for the NAD(P)H– Isokaempferide complex; and 23,860.72 Å^2^ for apo-COX-2 and 23,515.82 Å^2^ for the COX-2– Isokaempferide complex, as shown in Figs. 6 A–C. These slight reductions in SASA values upon ligand binding suggest decreased solvent exposure and increased hydrophobic core compactness. When interpreted alongside RMSD, RMSF, and Rg analyses, the SASA results further support that 5-LOX, NAD(P)H oxidase, and COX-2 adopt more stable and compact conformations when complexed with Isokaempferide.

#### Mechanism of binding interactions based on binding free energy calculations

To better understand the binding energetics of Isokaempferide with 5-LOX, NAD(P)H oxidase, and COX-2, the total binding free energy was computed using the Molecular Mechanics Generalized Born Surface Area (MM-GBSA) method implemented in AMBER14. Snapshots were extracted from the molecular dynamics trajectories to perform the energy calculations, and the results are summarized in Table 4. The analysis revealed distinct differences in the binding affinities of Isokaempferide across various targets. Notably, the binding free energy of Isokaempferide with EGFR was − 44.58 kcal/mol, compared to − 38.95 kcal/mol for its interaction with Tubulin. This suggests a more favorable and energetically stable binding of Isokaempferide toward EGFR, indicating its higher potential as an EGFR inhibitor relative to Tubulin.

**Table 4:** Binding free energies (in kcal/mol) of the 5-LOX–Isokaempferide, NAD(P)H–Isokaempferide, and COX-2–Isokaempferide complexes calculated using the MM-GBSA method from MD simulation trajectories.

Energy Components (kcal/mol)					
Complex	Δ E_vdW_	ΔE_elec_	ΔG_gas_	ΔG_solv_	ΔG_bind_
**5-LOX—**Isokaempferide	-30.57 ± 0.73	-13.69 ± 0.99	-44.26 ± 0.25	29.02 ± 0.31	-15.24 ± 0.99
**NAD(P)H-**Isokaempferide	-31.41 ± 0.79	-79.24 ± 0.24	-110.6 ± 0.70	59.47 ± 0.87	-51.19 ± 0.26
**COX-2-** Isokaempferide	-42.98 ± 0.50	-22.98 ± 0.60	-65.97 ± 0.40	30.10 ± 0.90	-35.87 ± 0.60

The decomposition of total binding free energy using the MM-GBSA method into individual energy components provided deeper insight into the molecular basis of the binding interactions. The analysis revealed that van der Waals interactions were the primary contributors to the favorable binding of Isokaempferide with 5-LOX and COX-2, highlighting the importance of hydrophobic contacts in stabilizing these complexes. In contrast, the NAD(P)H–Isokaempferide complex showed a relatively weaker contribution from van der Waals interactions. Additionally, the polar solvation energy term was found to contribute unfavorably across all complexes, acting as a destabilizing factor in the binding process.

#### Identification of key residues responsible for inhibitor binding

To elucidate the specific amino acid residues contributing to the inhibitory binding of Isokaempferide, the total binding free energy was further decomposed on a per-residue basis using MM-GBSA energy decomposition analysis. This approach enabled the identification of key residues that play critical roles in stabilizing the ligand–protein complexes.

**Fig. 7:**
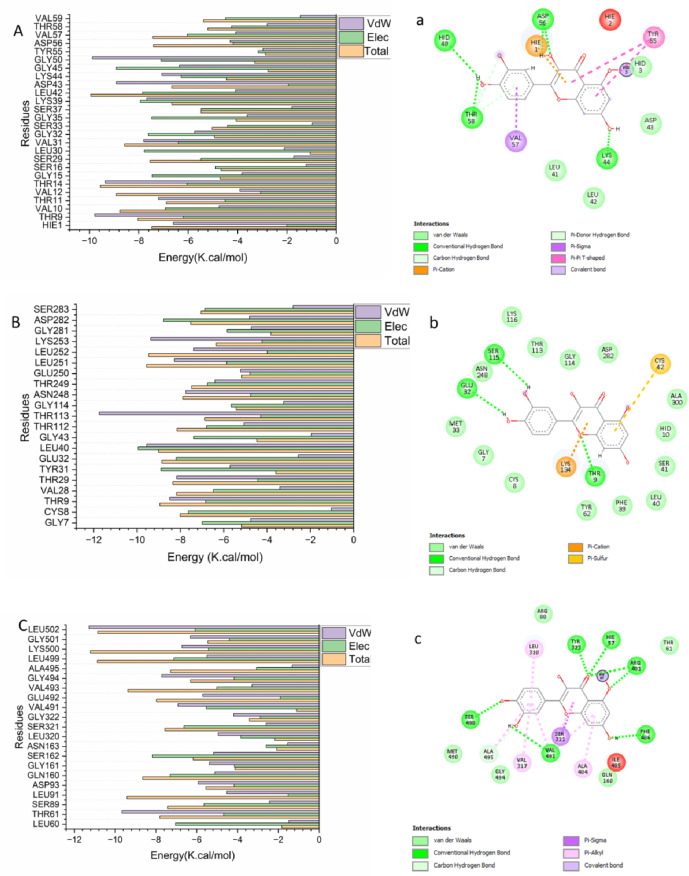
Per-residue decomposition energy plots illustrating the contributions of individual amino acid residues to the binding and stabilization of Isokaempferide at the catalytic sites of (**A**) 5-LOX, (**B**) COX-2, and (**C**) NAD(P)H oxidase. Corresponding ligand–protein interaction diagrams are shown in (**a**) 5-LOX, (**b**) COX-2, and (**c**) NAD(P)H, highlighting key residues involved in hydrogen bonding and hydrophobic interactions.

As shown in Fig. 7, several residues within the 5-LOX active site contributed significantly to the favorable binding of Isokaempferide, including HIE1 (− 6.599 kcal/mol), Val10 (− 4.747 kcal/mol), Thr11 (− 7.20 kcal/mol), Val31 (− 7.83 kcal/mol), and Gly50 (− 9.87 kcal/mol). For the NAD(P)H oxidase system, major contributions were observed from Thr9 (− 8.489 kcal/mol), Thr29 (− 8.171 kcal/mol), Thr113 (− 11.742 kcal/mol), Gly114 (− 3.229 kcal/mol), and Lys253 (− 9.367 kcal/mol). In the COX-2 complex, key interacting residues included Thr61 (− 9.663 kcal/mol), Asp93 (− 5.928 kcal/mol), Ser162 (− 5.173 kcal/mol), and Val491 (− 6.691 kcal/mol).

These findings indicate that hydrogen bonding, polar interactions, and hydrophobic contacts with these residues are essential for the stable binding of Isokaempferide within the active sites of all three targets.

#### Free energy landscape (FEL) analysis

**Fig. 8:**
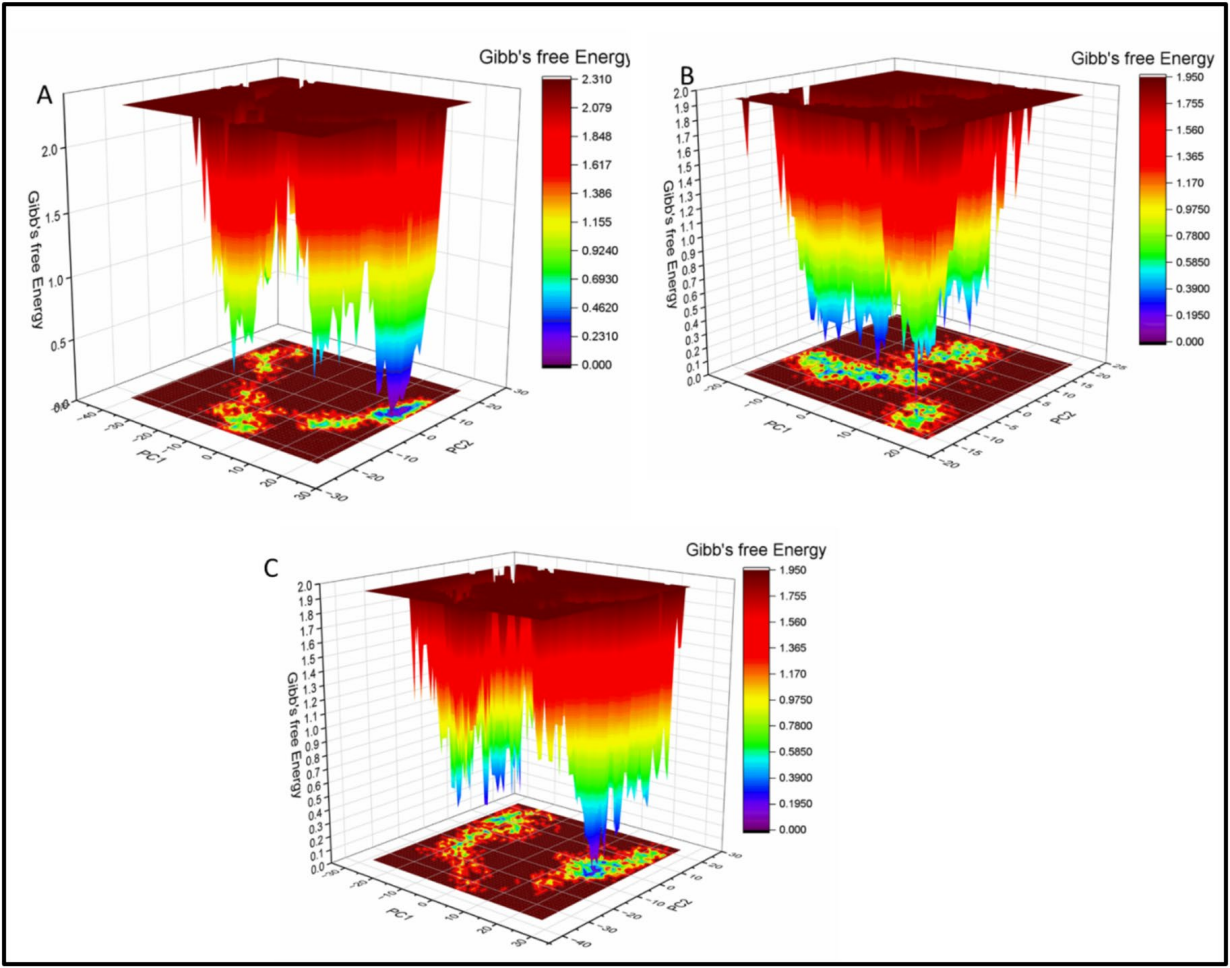
Free energy landscape (FEL) plots of Isokaempferide in complex with (**A**) 5-LOX, (**B**) COX-2, and (**C**) NAD(P)H oxidase. The landscapes were generated based on principal component analysis (PCA) of the MD trajectories, where low-energy conformational states are indicated by blue to violet regions, reflecting thermodynamically stable conformations.

Since protein conformational stability is closely associated with lower Gibbs free energy states, a principal component analysis (PCA)-based free energy landscape (FEL) analysis was conducted to evaluate the conformational stability of the simulated protein–ligand complexes. The resulting FEL plots are presented in Fig. 8. The FEL profiles revealed that the complexes occupied well-defined low-energy basins, indicating the formation of stable conformations throughout the simulation. These low-energy regions, represented by blue and violet zones in the landscape, suggest that the systems achieved thermodynamically favorable and structurally stable states during the MD trajectory.

#### Probability density function (PDF) analysis

**Fig. 9:**
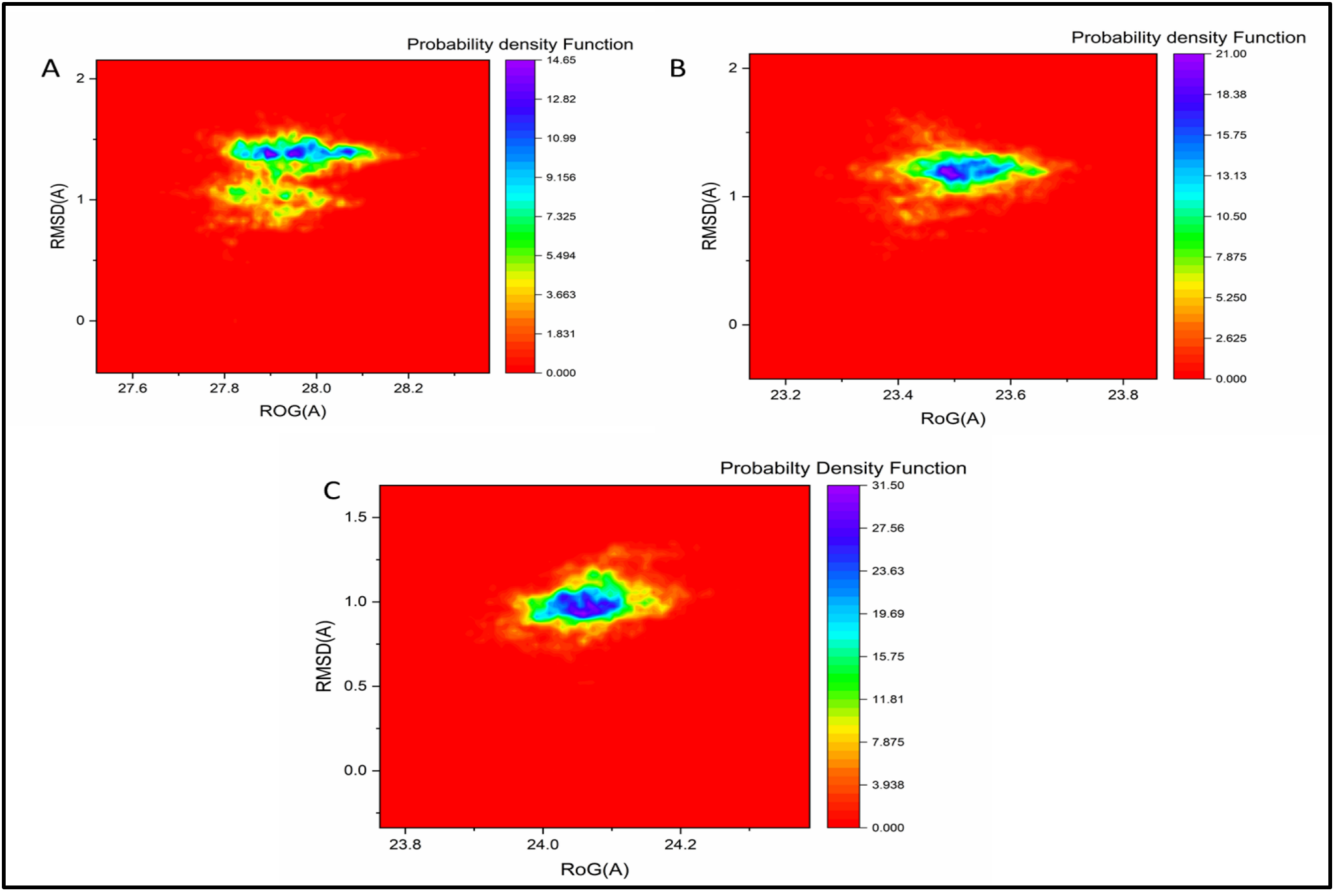
Probability density function (PDF) plots of Isokaempferide in complex with (**A**) 5-LOX, (**B**) COX-2, and (C) NAD(P)H oxidase. The plots are based on RMSD and radius of gyration (Rg) distributions over the MD simulation. Color gradients represent conformational populations, with red indicating the least populated and blue indicating the most populated structural states.

To further evaluate the conformational behavior and dominant structural states of the protein–ligand complexes, probability density function (PDF) analysis was performed using kernel density estimation (KDE)^60^**.** This approach provides insights into the most probable conformations sampled during the MD simulations, based on the distribution of RMSD and radius of gyration (Rg) values. As shown in Figs. 9 A–C, the PDF plots for each complex highlight the conformational states with the highest population density. For the 5-LOX– Isokaempferide complex, the most populated conformational state was observed at an Rg value of 27.59 Å and an RMSD value of 1.39 Å (Fig. 9A). In the case of the COX-2– Isokaempferide complex, the dominant conformer was centered around an Rg of 23.50 Å and an RMSD of 1.21 Å (Fig. 9B). Interestingly, the NAD(P)H– Isokaempferide complex exhibited its highest conformational density at an Rg of 24.05 Å and an RMSD of 0.97 Å (Fig. 9C). These results suggest that each protein–ligand complex stabilized into a well-defined conformational ensemble, with distinct structural preferences as reflected in their PDF peaks.

## Discussion

Plant-derived constituents are widely recognized for their diverse biological activities, including antioxidant, antimicrobial, antifungal, and anticancer effects, which are largely attributed to their rich phytochemical composition^54^**.** This broad pharmacological potential is intricately linked to the abundance and diversity of secondary metabolites, particularly flavonoids, which vary significantly across different subclasses. In this context, the quantitative determination of flavonoid subclasses in the flavonoids-enriched fraction of *Dodonaea viscosa* leaves demonstrated a pronounced dominance of flavones and flavonols (2518.6 ± 0.13 mg RE/100 g extract), compared to significantly lower concentrations of flavanones and dihydroflavonols (401.2 ± 0.11 mg NE/100 g extract). This chemical profile suggests that *D. viscosa* preferentially accumulates more oxidized flavonoid structures such as flavonols and flavones, which are known for their enhanced antioxidant capacity and broad pharmacological potential^61^**.** Such a pattern aligns with biosynthetic pathways that tend to favor the synthesis of flavonols over less oxidized forms like flavanones, possibly due to environmental pressures or genetic regulation^6^**.** These findings are strongly corroborated by previous phytochemical studies that have reported a wide spectrum of flavonoids in *D. viscosa*, including quercetin, kaempferol, and isorhamnetin—well-known representatives of the flavonol class^7,62^. Moreover, research has documented various bioactivities associated with these compounds, including antioxidant, anti-inflammatory, and hepatoprotective effects^63,64^. For instance, pinocembrin and other flavanones, though present in lower quantities, have also been linked to noteworthy biological effects, suggesting a synergistic contribution of both major and minor flavonoid constituents^61^. Taken together, the current results affirm the phytochemical richness of *D. viscosa* and underscore its potential as a natural source of bioactive flavonoids suitable for therapeutic development.

The LC–ESI–MS analysis of the flavonoids-enriched fraction from *Dodonaea viscosa* leaves unveiled a complex and diverse array of phytochemicals, notably phenolic acids and flavonoids, many of which exhibited distinctive and diagnostically valuable fragmentation behaviors. The identification of six phenolic acids—including p-coumaric acid, feruloylquinic acid, chlorogenic acid, and their esters and glycosides—was supported by characteristic decarboxylation, dehydration, and glycosidic bond cleavage pathways, producing fragments such as m/z 119, 135, 173, and 179, consistent with established fragmentation mechanisms for hydroxycinnamic acids^65,66^**.** Similarly, the identification of 16 flavonoids—spanning flavones, flavonols, flavan-3-ols, and their glycosides—highlighted the utility of retro-Diels–Alder (RDA) fragmentation, neutral losses (e.g., CO₂, CH₃), and interflavonoid bond cleavage in structural differentiation^50^. Key ions such as m/z 151, 179, 257, and 285 were instrumental in confirming aglycone identities, especially for kaempferol, quercetin, and their derivatives^67^**.** These fragmentation patterns align with previous studies on the structural elucidation of flavonoids and phenolic acids using tandem mass spectrometry^62,68^. These metabolomic insights not only underscore the phytochemical richness of *D. viscosa* but also provide a foundational understanding of the bioactive landscape contributing to its therapeutic potential. Building on this chemical profile, the flavonoid-enriched fraction of *Dodonaea viscosa* demonstrated strong antioxidant potential, as evidenced by its DPPH and ABTS radical scavenging activities. This observation is consistent with the findings of Malik (2022), who reported the highest antioxidant activity in the plant’s flowers, along with notable scavenging potential in its root and stem. The therapeutic significance of these results lies in the capacity of *D. viscosa* flavonoid-rich extracts to counteract oxidative stress, a process increasingly recognized as a critical factor in the onset and progression of pathological conditions such as inflammatory diseases, ischemic disorders, neurological disorders, hemochromatosis, emphysema, and acquired immunodeficiency syndrome^69^. These antioxidant properties are largely attributed to the abundance of phenolic and flavonoid compounds, which are well documented for their ability to neutralize free radicals, reduce oxidative stress, and enhance cellular antioxidant defenses (Rodríguez-Arce & Saldías, 2021). Further supporting this, Auta et al.^54^ demonstrated the protective role of *D. viscosa* stem and leaf extracts against oxidative stress, while Riaz et al.^48^ established a linear relationship between antioxidant potency, radical scavenging efficiency, and the phenolic content of *D. viscosa* leaves. Collectively, these findings highlight the potential application of *D. viscosa* extracts in fields such as food preservation and cosmetic formulations, where their antioxidant capacity can be harnessed to improve product stability and confer health-promoting benefits.

Interestingly, the same phytochemicals implicated in antioxidant activity also contribute to the extract’s pronounced anti-inflammatory effects. The dual inhibition of COX-2 and 5-LOX enzymes by the flavonoid-rich fraction highlights a synergistic mechanism of action, potentially reducing inflammation through multiple signaling pathways. The observed activity can be attributed to the high flavonoid content in the extract, as flavonoids are known to exert anti-inflammatory effects during both proliferative and exudative stages of inflammation by targeting key enzymes such as nitric oxide synthase, xanthine oxidase, LOX, and COX^70^. This dual functionality not only expands the pharmacological relevance of *D. viscosa*, but also supports its application in the management of oxidative stress-related inflammatory disorders.

Structurally, flavonoids possess a benzene ring (A) fused with a six-membered ring (C) and a phenyl ring (B) at the 2-position^71^, enabling specific interactions with inflammatory enzyme targets. Their mechanism of action involves hydrogen bonding with the active sites of COX, π–π stacking with the Tyr355 residue at the COX entrance, and hydrophobic interactions within the enzyme’s binding pocket. In the case of 5-LOX inhibition, hydrogen bonding with the Ala424 residue has been proposed as a key interaction^72^. Supporting evidence from earlier studies further underscores the therapeutic promise of *D. viscosa*. For instance, viscosine, a flavonoid isolated from this species, demonstrated strong binding affinity for both COX-2 and 5-LOX in molecular docking studies^73^**.** Additionally, hautriwaic acid, a diterpene also derived from *D. viscosa*, significantly inhibited inflammation in a TPA-induced mice ear edema model, achieving up to 87.1% inhibition at 1.0 mg/ear—surpassing the efficacy of indomethacin at comparable doses^74^. Furthermore, the hydroalcoholic extract of *D. viscosa* has been shown to reduce carrageenan-induced paw edema in rats, lending in vivo support to its anti-inflammatory efficacy^75^. Collectively, these findings reinforce the value of *D. viscosa* as a promising natural source of dual COX-2 and 5-LOX inhibitors with potential therapeutic applications.

To connect these experimental findings with computational insights, preliminary docking analyses were performed on several of the identified flavonoids and phenolic acids. Among them, isokaempferide demonstrated the most favorable binding affinities and stable interaction profiles across COX-2, 5-LOX, and NAD(P)H oxidase. This, together with its relative abundance in the fraction and prior reports of biological activity, justified its selection for detailed molecular docking and molecular dynamics (MD) simulations. Despite these promising results, it is important to acknowledge that the present findings are based on fraction-level analyses and computational predictions. Future studies focusing on the purification and bioassay-guided evaluation of individual compounds will be essential to validate their specific contributions to the observed antioxidant and anti-inflammatory activities.

The MD results reinforced its potential, showing that isokaempferide maintained stable binding conformations with consistently low RMSD values, reduced residue flexibility (RMSF), compact protein–ligand structures (Rg), and favorable MM-GBSA binding energies dominated by van der Waals interactions. Collectively, these computational outcomes suggest that isokaempferide forms robust and stable interactions within the catalytic pockets of key pro-inflammatory enzymes, supporting its role as a multifunctional inhibitor. Nevertheless, it should be emphasized that the current findings are based on fraction-level experimental assays and computational predictions of selected compounds. Future work should focus on the isolation and bioassay-guided testing of individual metabolites to directly validate their contributions to the observed antioxidant and anti-inflammatory effects.

## Conclusion

This study demonstrates the rich phytochemical diversity and notable pharmacological potential of the flavonoid-enriched fraction derived from *Dodonaea viscosa* leaves. LC–ESI–MS profiling and enzyme inhibition assays confirmed a predominance of flavones and flavonols, which correlate with the observed antioxidant and anti-inflammatory activities. Mechanistic insights obtained from molecular docking and molecular dynamics simulations highlighted the multi-targeted interactions of these flavonoids. In particular, isokaempferide exhibited high structural stability and compactness within protein–ligand complexes, while MM-GBSA binding free energy calculations further supported its strong and stable interactions with 5-LOX, NAD(P)H oxidase, and COX-2. To the best of our knowledge, this is the first study to identify isokaempferide as a promising dual antioxidant and anti-inflammatory candidate, providing novel insights for future drug discovery and development. Nonetheless, as the present findings are based on fraction-level analysis and computational predictions, definitive conclusions regarding the precise compounds directly responsible for the observed biological activities remain limited. Future investigations should therefore extend computational analyses to additional identified compounds and prioritize purification and bioassay-guided evaluation of individual flavonoids to validate and expand these findings, thereby facilitating the development of *D. viscosa*–derived therapeutic, nutraceutical, and functional applications.

## Supplementary Information

Supplementary Information.

## Data Availability

The datasets used and/or analysed during the current study available from the corresponding author on reasonable request.
