# Supplementary material for: Computational and bioactivity investigations of flavonoid fraction from Dodonaea viscosa against oxidative stress and inflammation
Source: Sci Rep. 2025 Dec 11;15:43652. doi: 10.1038/s41598-025-29576-0 (PMC12701060; doi:10.1038/s41598-025-29576-0)
Supplement: Supplementary file 1 — Supplementary Information. [file 41598_2025_29576_MOESM1_ESM.docx]

**Computational and Bioactivity Investigations** **of Flavonoid Fraction from Dodonaea viscosa Against Oxidative Stress and Inflammation**

# Amal M. El-Feky ^1*^, Ahmed A. El-Rashedy ^2,3^, Noha E. Ibrahim ^4^

# ^1^ Pharmacognosy Department, National Research Centre, 33 El Bohouth St. (Former El Tahrir St.), Dokki, P.O. 12622, Giza, Egypt. ^2^ Chemistry of Natural and Microbial Products Department, National Research Center (NRC), Egypt. ^3^ Department Organic and Medicinal Chemistry, Faculty of Pharmacy, University of Sadat City, Menoufia, 32897, Egypt. ^4^ Microbial Biotechnology Department, Biotechnology Research Institute, National Research Centre, 33 El Bohouth St. (Former El Tahrir St.), P.O. 12622, Dokki, Giza, Egypt.

**For Correspondence:* Amal M. El-Feky, [*ammelfeky@hotmail.com*](mailto:ammelfeky@hotmail.com)

**Supplementary Table S1:** Autodocking Vina docking results for Extracted compounds docked into the catalytic domain binding site of 5-LOX , COX-2, NAD(P)H. receptor in comparison to the co-crystallized ligand

| **Compounds** | **Hydrogen bonds between atoms of compounds and amino acids of receptor** | | | | | | | | **S- score**  **(binding energy) (kcal/mol)** |
| --- | --- | --- | --- | --- | --- | --- | --- | --- | --- |
|  | **Compounds** | **receptor** | | | **Type** | | **Distance (Å)** | |  |
|  | **Atoms** | **Atoms** | **Residues** | |  |  |  |  |  |
| **5-LOX** | | | | | | | | | |
| *P*-Coumaric acid | H 10260 | O 10925 | Val 50 | | H-donor | | 1.78 | | -9.39 |
|  | H 10217 | O 10953 | Ser 50 | | H-donor | | 1.99 | |  |
| Feruloyl quinic acid | H 10260 | O 10925 | VAL 50 | | H-donor | | 1.78 | | -8.34 |
|  | H10217 | O10925 | Ser 52 | | H-donor | | 1.99 | |  |
| Caffeoyl-O-hexoside | H10260 | O10925 | Val50 | | H-donor | | 1.78 | | -8.82 |
|  | H10217 | O10963 | Ser 52 | | H-donor | | 1.99 | |  |
| Chlorogenic acid | H10260` | O10925 | Val 50 | | H-donor | | 1.78 | | -9.19 |
| *p*-Coumaric acid ethyl ester | H10260 | O10925 | Val50 | | H-donor | | 1.99 | | -7.89 |
| Myricetin-*O*-hexoside | H10260 | O10925 | Val50 | | H-donor | | 1.78 | | -9.14 |
| Coumaroyl-*O*-caffeoylquinic acid | H10260 | O10925 | Val50` | | H-donor | | 1.78 | | -9.17 |
| Quercetin-*O*-hexoside | H10217 | O10953 | Ser 52 | | H-donor | | 1.99 | | -9.36 |
| 5,7,4-Trihydroxy-3-(4-hydroxy 3-Methylbutyl)-5-prenyl-3, 6-diMethoxyflavone | H10217 | O10953 | Ser52 | | H-donor | | 1.99 | | -8.97 |
| Kaempferol-*O*-rutinoside | H10225 | O10953 | Ser50 | | H-donor | | 1.94 | | -9.15 |
| Quercetin-*O*-pentoside | H10217 | O10653 | Ser 52 | | H-donor | | 1.94 | | -9.67 |
| Viscosol | H10276 | OG10957 | Ser52 | | H-donor | | 1.94 | | -9.23 |
| Catechin | No interaction |  |  | |  | |  | |  |
| Rutin | No interaction |  |  | |  | |  | |  |
| Kaempferol-*O*-rhamnoside | H10276 | OG10957 | Ser52 | | H-donor | | 2.88 | | -10.98 |
| Quercetin 3' -*O*-methyl ether | H10260 | O10925 | Val50 | | H-donor | | 1.78 | | -9.08 |
| 3,5-Dihydroxy-4' ,7-dimethoxyflavone | H10217 | O10953 | Ser52 | | H-donor | | 1.99 | | -8.35 |
| 5,7,4' ,5' -Tetrahydroxy-3,6,2'– trimethoxyflavone | H10225 | O10953 | Ser52 | | H-donor | | 1.94 | | -8.16 |
| 5,7-Dihydroxy-3,6,4'-trimethoxyflavone | H10217 | O10953 | Ser52 | | H-donor | | 1.99 | | -8.49 |
| Kaempferol | No interaction |  |  | |  | |  | |  |
| Isokaempferide | H10260 | O10925 | Val50 | | H-donor | | 1.78 | | -11.06 |
|  | H10217 | O10953 | Ser50 | | H-donor | | 1.99 | |  |
|  | H10255 | O10953 | Ser52 | | H-donor | | 1.94 | |  |
|  | N10276 | OG10957 | Ser52 | | H-donor | | 2.88 | |  |
| Quercetin | No interaction |  |  | |  | |  | |  |
| **NAD(P)H** | | | | | | | | | |
| *P*-Coumaric acid | no interaction |  |  |  | |  | |  | |
| Feruloyl quinic acid | O13877 | OG606 | Ser41 | H-donor | | 2.29 | | -10.02 | |
| Caffeoyl-O-hexoside | O13873 | OH2919 | Tyr188 | H-donor | | 3.00 | | -10.42 | |
| Chlorogenic acid | H13901 | OE2513 | Glu163 | H-donor | | 2.52 | | -10.53 | |
| *p*-Coumaric acid ethyl ester | No interaction |  |  |  | |  | |  | |
| Myricetin-*O*-hexoside | O13877 | OG606 | Ser41 | H-acceptor | | 2.48 | | -11.90 | |
| Coumaroyl-*O*-caffeoylquinic acid | O13910 | N2759 | Gly 180 | H-acceptor | | 2.36 | | -7.39 | |
| Quercetin-*O*-hexoside | H19334 | OD4364 | Asp282 | H-acceptor | | 2.11 | | -9.48 | |
| 5,7,4-Trihydroxy-3-(4-hydroxy 3-Methylbutyl)-5-prenyl-3, 6-diMethoxyflavone | H13936 | O4586 | Pro298 | H-donor | | 2.46 | | -11.97 | |
| Kaempferol-*O*-rutinoside | No interaction |  |  |  | |  | |  | |
| Quercetin-*O*-pentoside | No interaction |  |  |  | |  | |  | |
| Viscosol | O13890 | NZ2065 | Lys134 | H-acceptor | | 2.57 | | -9.78 | |
| Catechin | O13886 | OD618 | Cys 42 | H-donor | | 2.61 | | -11.31 | |
| Rutin | O13970 | OG606 | Ser41 | H-donor | | 2.62 | | -9.89 | |
| Kaempferol-*O*-rhamnoside | No interaction |  |  |  | |  | |  | |
| Quercetin 3' -*O*-methyl ether | No interaction |  |  |  | |  | |  | |
| 3,5-Dihydroxy-4' ,7-dimethoxyflavone | No interaction |  |  |  | |  | |  | |
| 5,7,4' ,5' -Tetrahydroxy-3,6,2'– trimethoxyflavone | H13905 | OD4364 | Asp282 | H-donor | | 1.80 | | -10.54 | |
| 5,7-Dihydroxy-3,6,4'-trimethoxyflavone | H13895 | OE2511 | Glu163 | H-donor | | 2.88 | | -13.61 | |
| Kaempferol | H13896 | O5029 | Ser327 | H-donor | | 2.87 | | -10.27 | |
| Isokaempferide | H13895 | O563 | Phe 39 | H-donor | | 3.35 | | -16.01 | |
|  | O13885 | OD618 | Cys42 | H-donor | | 2.95 | |  | |
|  | O13885 | N597 | Ser41 | H-acceptor | | 2.75 | |  | |
| Quercetin | No interaction |  |  |  | |  | |  | |
| **COX-2** | | | | | | | | | |
| *P*-Coumaric acid | No interaction |  |  |  | |  | |  | |
| Feruloyl quinic acid | H8858 | O7904 | Met522 | H-donor | | 3.25 | | -11.21 | |
| Caffeoyl-O-hexoside | O8836 | OH5163 | Tyr355 | H-donor | | 3.06 | | -11.31 | |
| Chlorogenic acid | O8835 | NE862 | His90 | H-donor | | 2.70 | | -9.39 | |
| *p*-Coumaric acid ethyl ester | No interaction |  |  |  | |  | |  | |
| Myricetin-*O*-hexoside | O8846 | OH5136 | Tyr355 | H-donor | | 2.68 | | -11.70 | |
| Coumaroyl-*O*-caffeoylquinic acid | No interaction |  |  |  | |  | |  | |
| Quercetin-*O*-hexoside | H8877 | OE7942 | Glu524 | H-donor | | 2.38 | | -8.18 | |
| 5,7,4-Trihydroxy-3-(4-hydroxy 3-Methylbutyl)-5-prenyl-3, 6-diMethoxyflavone | H8885 | OE2516 | Gln192 | H-donor | | 1.12 | | -6.34 | |
| Kaempferol-*O*-rutinoside | O8851 | OH5163 | Tyr355 | H-donor | | 2.63 | | -6.47 | |
| Quercetin-*O*-pentoside | No interaction |  |  |  | |  | |  | |
| Viscosol | H8858 | OE2516 | Gln192 | H-donor | | 1.20 | | -9.66 | |
| Catechin | O8833 | OH5163 | Tyr355 | H-donor | | 2.35 | | -11.46 | |
| Rutin | O8852 | ND857 | His90 | H-donor | | 2.41 | | -7.00 | |
| Kaempferol-*O*-rhamnoside | No interaction |  |  |  | |  | |  | |
| Quercetin 3' -*O*-methyl ether | No interaction |  |  |  | |  | |  | |
| 3,5-Dihydroxy-4' ,7-dimethoxyflavone | O8833 | OG8021 | Ser530 | H-acceptor | | 2.36 | | -11.92 | |
| 5,7,4' ,5' -Tetrahydroxy-3,6,2'– trimethoxyflavone | O8834 | OG8021 | Ser 530 | h-acceptor | | 2.49 | | -12.33 | |
| 5,7-Dihydroxy-3,6,4'-trimethoxyflavone | H8841 | OE2516 | Gln192 | H-donor | | 3.17 | | -12.35 | |
| Kaempferol | No interaction |  |  |  | |  | |  | |
| Isokaempferide | O8833 | OH5163 | Tyr355 | H-donor | | 2.7 | | -15.78 | |
|  | O8823 | ND857 | His 90 | H-acceptor | | 2.66 | |  | |
|  | O8833 | OH5163 | Tyr355 | H-acceptor | | 2.75 | |  | |
| Quercetin | No interaction |  |  |  | |  | |  | |
